# Fungal endophytes of *Plumbago zeylanica* L. enhances plumbagin content

**DOI:** 10.1186/s40529-019-0270-1

**Published:** 2019-09-07

**Authors:** Namdeo B. Andhale, Mohd. Shahnawaz, Avinash B. Ade

**Affiliations:** 1 Department of Botany, Savitribai Phule Pune University, Ganeshkhind, Pune, Maharashtra 411007 India; 2 Department of Biology, Fergusson College, FC Road, Shivajinagar, Pune, MS 411004 India; 3 Present Address: Plant Biotechnology Division, CSIR-Indian Institute of Integrative Medicine, Canal Road Jammu, Jammu, J&K 180001 India

**Keywords:** *Plumbago zeylanica*, Secondary metabolites, Plumbagin, Endophytic fungi, Enhancement

## Abstract

**Background:**

Plumbagin is one of the pharmaceutically important biomolecule with anticancer potential. Among the plants reported to produce plumbagin, *P. zeylanica* topped the list. The plumbagin production is very slow with low yield and maximum 0.5% (of dry weight) was reported in *P. zeylanica*. To meet the increasing demand of the plumbagin at global level, the *P. zeylanica* are exploited at commercial level, which may pose serious threat on the germplasm of the plant populations. So, it is needed to enhance the contents of plumbagin in *P. zeylanica* using biotechnological approaches. Among the various methods used to enhance the contents of plumbagin in *P. zeylanica,* utilization of fungal endophytes to enhance the plumbagin contents is a widely accepted approach. As fungal endophytes have the potential to synthesize various secondary metabolites and also reported to influence the synthesis of the secondary metabolites in plants. In the present study, an attempt was made to assess the effect of fungal endophytes of the *Plumbago zeylanica* L. on enhancement of plumbagin contents at in vivo level.

**Results:**

Total 3 fungal endophytes were recorded from the roots of *P. zeylanica* collected from Khadki, Pune. The fungal endophytes were identified at morphological and molecular level. After 1 year of the treatment with fungal endophytes, significant enhancement of plumbagin was recorded in the roots of the *P. zeylanica*. Plumbagin contents in each were quantified against the standard plumbagin by employing LCMS-MS technique. Among the three fungal endophytes, the maximum enhancement of plumbagin content (122.67%) was reported with the treatment of *Alternaria  * sp. (Isolate-3) in the roots of the *P. zeylanica* compared to control.

**Conclusion:**

Among the three fungal endophytes, the maximum enhancement of plumbagin content (122.67%) was reported with *Alternaria * sp. (Isolate 3) in the roots of the pot-grown plants of *P. zeylanica* at in vivo level.

## Background

*Plumbago zeylanica* L. (leadwort) is a herbaceous plant belongs to the family Plumbaginaceae and is reported as one of the potential multipurpose medicinal plant (Pant et al. 2012). It is native of South Asia and is distributed throughout the tropical and subtropical regions of the globe (Jain et al. 2014). The phytochemical analysis of the plant (*P. zeylanica*) extract revealed the presence of various secondary metabolites viz. 1,2 (3)-Tetrahydro-3,3′-biplumbagin, 1,4-naphthoquinone, 6-hydroxyplumbagin, alkaloid benzene, alkenes, anthocyanin pigments, benzenoid, beta-stigmasterol, campesterol, flavonoid, leucodelphinidin, lignoceric acid, naphthoquinone, naphthalenone, naphthylamine, phenolic acids, plumbagin, quinoid, sitosterol, and tannins in different parts of the plant (Jalalpure 2011; Ming et al. 2011; Zhang et al. 2007; Gunaherath et al. 1983). Plumbagin is reported as the active principle of the *P. zeylanica* (Tilak et al. 2004). Since ancient times, various medicinal potentials were attributed to *P. zeylanica*, in both Indian and Chinese systems of medicine (Patwardhan et al. 2004). Based on its phytochemical potentials, it is reported to have anti anorexic, anti-cancer, anti-dyspepsia, anti-haemorrhoidal, anti-saturative, appetizer, analgesic and antimicrobial activities (Vishnukanta and Rana 2010; Vijver and Looter 1971; Ahmad et al. 1998; Liu et al. 2017). The roots of the *Plumbago* sp. are the main source of Plumbagin (Komaraiah et al. 2002). Plumbagin (C_11_H_8_O_3_) is one of the most important secondary metabolites belong to 1,4-naphthoquinone class of the quinone family, with a molecular weight 188.18 g/mol (Liu et al. 2017). Plumbagin is also reported to be produced by various plant genera belongs to other botanical families viz. Droseraceae and Ebenaceae etc (Padhye et al. 2012) but the members of the family Plumbaginaceae reported to produce plumbagin at high concentration with maximum (1.0% of the dry weight) by *P. zeylanica* (Dohare et al. 2015). As per reports (Paiva et al. 2003; Sinha et al. 2013), plumbagin is having anticancer, antibacterial and antifungal activities (Aziz et al. 2008). Due to its prime importance, it has tremendous demand in various pharmaceutical companies (Mukherjee 2017). To meet the increasing demand of the plumbagin at global level, the *P. zeylanica* plants were exploited at commercial level, which may pose serious threat on the germplasm of the plant populations. Furthermore, the rate of plumbagin synthesis under natural condition is very slow due to variability in the species and changing environmental conditions (Roy and Bharadvaja 2018). So it is needed to develop the efficient, cost effective and accepted approach to enhance the contents of plumbagin. In past various people attempted to enhance the contents of the Plumbagin in *Plumbago* sp. using various methods e.g., generation of transgenic lines (Martin et al. 2011), elicitation and in situ adsorption (Komaraiah et al. 2002; Jaisi and Panichayupakaranant 2016), production of mutants using induced mutation (Jaisi et al. 2013), developing root suspension culture (Roy and Bharadvaja 2019), triggering the pathways using bacterial treatment to the roots at in vivo level (Andhale and Ade 2013), and treatment of plant roots with fungal endophytes (Pandey et al. 2016). Among all the methods, the technique which employed fungal endophytes, seems cost-effective, accepted and efficient method to enhance secondary metabolite contents in the plants. So, in the present study an attempt was made to study the effect of fungal endophytes of *P. zeylanica* on its own plumbagin contents at in vivo level.

## Methods

### Collection, identification and authentication of the *P. zeylanica* L

The *P. zeylanica* plants were collected randomly from three to four locations along road sides of Park Road (18° 56′ 06.5″ N; 73° 83′ 91.6″ E), QMTI quarters (near the Ayyappa temple), Khadki, Pune, Maharashtra-411045. The voucher specimen of the plant was also submitted at Botanical Survey of India (BSI), Western Circle, Pune for identification and authentication.

### Isolation of fungal endophytes

Fungal endophytes from the roots of *P. zeylanica* were isolated using the method of Hallmann et al (2006). The roots were separated from the plant and washed with tap water followed by surface sterilization using 0.1% HgCl_2_ for 3 min and 70% C_2_H_5_OH for 1 min. The surface sterilized roots were rinsed with autoclaved water and dried using sterile tissue paper under aseptic condition. The roots were chopped with sterile scalpel into small pieces (1 × 1 mm^2^). Sterilized root pieces were inoculated on plain agar plates, incubated with at 27 ± 2 °C for 2 days under dark condition. Single mycelia developed from each root was inoculated on each Potato Dextrose Agar (PDA) plate (Potato Dextrose Agar, Cat. No. M096, Himedia) supplemented with streptomycin 100 μg/ml was used). PDA inoculated plates were incubated (Incubator, Steel Mate Novatech, Pune)  at 27 ± 2 °C for 15 days under dark condition. After the growth the endophytic fungi, the pure cultures were separated and sub-cultured on Rose Bengal Agar (Rose Bengal Agar media, Cat. No. M842, Himedia) and plates were incubated with at 27 ± 2 °C for 15 days under dark condition.

### Identification of fungal endophytes

The pure cultures of the fungal endophytes were identified at morphological and molecular level.

### Morphological Identification of the fungal endophytes

At morphological level, all the fungal endophytes were identified based on morphological keys at National Fungal Culture Collection of India (FCCI), Agharkar Research Institute (ARI), Gopal Ganesh Agarkar Road, Pune, Maharashtra 411004, India.

### Molecular identification of the fungal endophytes

Fungal endophytes with potential to enhance the plumbagin content were only identified at molecular level.

#### Extraction of genomic DNA

Total genomic DNA was extracted from the top 2 elite fungal endophytes (7 to 14 days old culture) grown on potato dextrose agar (PDA) media (Himedia, Mumabai) homogenized in a FastPrep 24 tissue homogenizer (MP Biomedicals, Germany) using the salt extraction method (Aljanabi and Martinez 1997). The quality and quantity the extracted DNA was checked using 0.8% Agarose (Sigma-Aldrich, Missouri, United States) gel electrophoresis (1× TAE buffer) stained with ethidium bromide (10 µg/ml) against the standard λ DNA. The gel documentation was done using gel documentation unit (Alpha Imager, Germany). The concentration of the DNA was also determined using Nanodrop (NanoDrop, 800, Thermo Scientific, USA) and finally the concentration of DNAs were diluted to 10 ng/µl for PCR amplification.

#### Amplification of ITS gene

The universal Internal transcribed spacer (ITS) gene was amplified using ITS4 and ITS5 primers (White et al. 1990). Total 25 µl volume of polymerase chain reaction (PCR) mixture comprises of 16 μl PCR grade water (Sigma), 2.5 μl PCR buffer (10×); 1 μl dNTPs (250 mM each); 0.5 μl of each primer (50 pmol/μl); 1 μl (1 U/μl) of Taq polymerase (all from Bangalore Genie, Bangalore, India) along with 2.5 μl (10 ng/μl) of template DNA and was overlaid with one drop of light mineral oil. The amplification of the ITS gene was performed in a thermocycler (Eppendorf MasterCycler, Hamburg, Germany) and was programmed with initial denaturation temperature at 95 °C for 5 min, followed by 30 cycles of denaturation at 95 °C for 1 min, annealing at 56 °C for 1 min and extension at 72 °C for 1 min. The final extension was done at 72 °C for 7 min. The PCR products obtained were electrophoresed using 1.5% Agarose gel stained with ethidium bromide along with 100 bp DNA ladder (Fermentas, USA), followed by gel documentation using gel documentation unit (Alpha Imager, Germany).

#### Elution and sequencing of the PCR products

The amplified band was eluted using QIAquick Gel Extraction Kit (QIAGEN GmbH, Germany) by following the instruction manual. The eluted bands were further quantified using both 1.5% Agarose gel electrophoresis and nanodrop before sequencing. The eluted solutions were sequenced using Big Dye Terminator cycle sequencing kit (Applied Biosystems, Foster City, CA) as per the manufacturer’s instructions on an ABI 3100 Avant Prism automated DNA sequencer (Applied Biosystems). The raw sequences obtained were viewed using ChromasLite v. 2.01 (http://www.technelysium.com.au) and curated manually.

#### Molecular characterization of the elite fungal endophytes and construction of dendrogram

The evolutionary history was inferred using the Neighbor-Joining method (Saitou and Nei 1987). The optimal tree with the sum of branch length = 0.35543798 is shown. The percentage of replicate trees in which the associated taxa clustered together in the bootstrap test (1000 replicates) are shown next to the branches (Felsenstein 1985). The evolutionary distances were computed using the Maximum Composite Likelihood method (Tamura et al. 2004) and are in the units of the number of base substitutions per site. The analysis involved 18 nucleotide sequences. Codon positions included were 1st + 2nd + 3rd + Noncoding. All positions containing gaps and missing data were eliminated. There were a total of 484 positions in the final dataset. Evolutionary analyses were conducted in MEGA6 (Tamura et al. 2013).

### Submission of the fungal endophytes to culture collection repository

The pure cultures of the two efficient plumbagin enhancing fungal endophytes were submitted at National Fungal Culture Collection of India (FCCI), Agharkar Research Institute (ARI), Gopal Ganesh Agarkar Road, Pune, Maharashtra 411004, India for general deposition and were accessioned.

### Experimental setup and fungal endophytic treatments to enhance plumbagin contents

The randomized block design was used for the experiment. Total four sets (using pots) in triplicate were made using sterile soil to grow the plants of *P. zeylanica* L. using different treatments. All the plants were grown in respective pots were kept at Botanical Garden, Department of Botany, Savitribai Phule Pune University, Pune. To Set-1 distilled water was added and it served as a control, furthermore, the other sets (Set-2, Set-3 and Set-4) were inoculated with the endophytic fungal (EF) inoculum (A suspension of 10 ml sterile water + Loop full of endophytic fungus culture) of the respective fungal endophytes (fungal endophyte 1, fungal endophyte 2 and fungal endophyte 3) once a month for a period of 12 months.

### Harvesting of the roots for extraction of plumbagin

After completion of the 12 months of treatment, roots of the *P. zeylanica* were harvested to quantify the contents of plumbagin using Liquid Chromatography Mass Spectrometry-Mas Spectra (LCMS-MS) technology.

### Sample preparation for LCMS-MS analysis

Samples for the LCMS-MS analysis were prepared using the protocol of Hsieh et al. (2005) with desired modifications. 10 g roots of *P. zeylanica* were powdered and homogenized in 50 ml Tarson tubes with 10 ml methanol (HPLC grade, Merck, Darmstadt, Germany) and were sonicated for 10 min followed by centrifugation at 5000 rotations per minutes (rpm) for 10 min at 20 °C. The methanolic root extract was further filtered by passing through sieve of 0.40 µm size. The methanolic root extract of the plants were further used to quantify the plumbagin content using the LCMS-MS compared to the standard (plumbagin).

### Preparation of standard plumbagin solution

1 mg/ml stock solution of the plumbagin powder (Hi-Media, Mumbai, India) using methanol (HPLC grade, Merck, Darmstadt, Germany) was made. Furthermore, the stock solution was diluted using methanol to get various working concentrations (0.1, 0.2, 0.5 and 1 μg/ml) of the standard plumbagin, respectively (Pawar et al. 2011).

### LCMS-MS analysis of the methanolic root extracts

LCMS/MS is considered as most robust and sensitive technique to detect analytes at faster rate (Gallardo et al. 2009). The LCMS-MS analysis was executed at TUV Nord India Pvt. Ltd., Branch Baner, Pune. In the present study UPLCMS system ACQUITY UPLC I-Class (Waters, USA) was used to detect the plumbagin content in the methanolic root extract of the *P. zeylanica*. The UPLCMS system was equipped with a flow-through-needle injector design (SM-FTN) to accommodate the highest range of injection volume, to maximize recovery of the tested samples and to achieve the precision in the performance of the injection (Annonymous 2010). The ACQUITY BEH C18 (2.1 × 100 mm, 1.7 µm) column compartment of the UPLCMS system was coupled with Triple quadrupole mass spectrometer (SYNAPT G2-S HDMS, WATERS, USA) having with a positive ESI mode of detection. The mobile phase of solvent A (100% water) and solvent B (100% methanol) used for gradient elution with [0–0.00 min (60% A, 40% B), 3.0 min (linear gradient to 40% B), 3.00–5.00 min (80% A, 20% B), 5 min (linear gradient to 80% A), 5.00–8.00 min (60% A, 40% B), 8 min (linear gradient to 60% A)] at a flow rate of 0.300 ml/min. The temperature of column oven was maintained at 45 °C, and the injection volume was 5 μl. The conditions of MS system was operated at positive ESI mode and the ions were detected using multiple reactions monitoring (MRM) mode (Additional file 1: Table S1) with 150 °C gas temperature, 1000 l/h gas flow rate and 550 °C as gas sheath temperature.

### Data processing and analysis

The data files of the LCMS-MS analysis were obtained using Waters’ Mass Lynx Software. The generated data was further processed and analysed using Target Lynx Software.

### Calculation of percent enhancement of the plumbagin

The percentage of the plumbagin enhancement was determined using the given formula:

$${\text{\% EP}} = \frac{{{\text{Amount of plumbagin }}\left( {\text{ppm}} \right) {\text{in the TP}} - {\text{Amount of plumbagin }}\left( {\text{ppm}} \right){\text{in the CP}}}}{{{\text{Amount of plumbagin }}\left( {\text{ppm}} \right){\text{in CP}}}} \times 100$$

whereas, % EP: percent enhancement of plumbagin, CP: control plants, TP: treated plants.

### Statistical analysis

All the treatments were in triplicates. The means and standard deviation to the mean values were calculated using MS Excel, 2010. To test the significance of the results at 0.05 level of significance, one-way analysis of variance (ANOVA) was performed using SPSS Inc software (SPSS 16, 2017).

## Results

### Identification of the collected *P. zeylanica*

The collected plant accession of the *Plumbago* sp. was identified and authenticated at Botanical survey of India (BSI) Western Circle Pune (No. BSI/WRC/Cert./2015) as *Plumbago zeylanica* L.

### Isolation of the fungal endophytes

Only three fungal endophytes (fungal endophytes 1, fungal endophytes 2 and fungal endophytes 3) were reported from the collected roots of the *P. zeylanica.*

## Estimation of plumbagin contents in *P. zeylanica* L. after the treatment of fungal endophytes

### The percent increment of the plumbagin

**Fig. 1:**
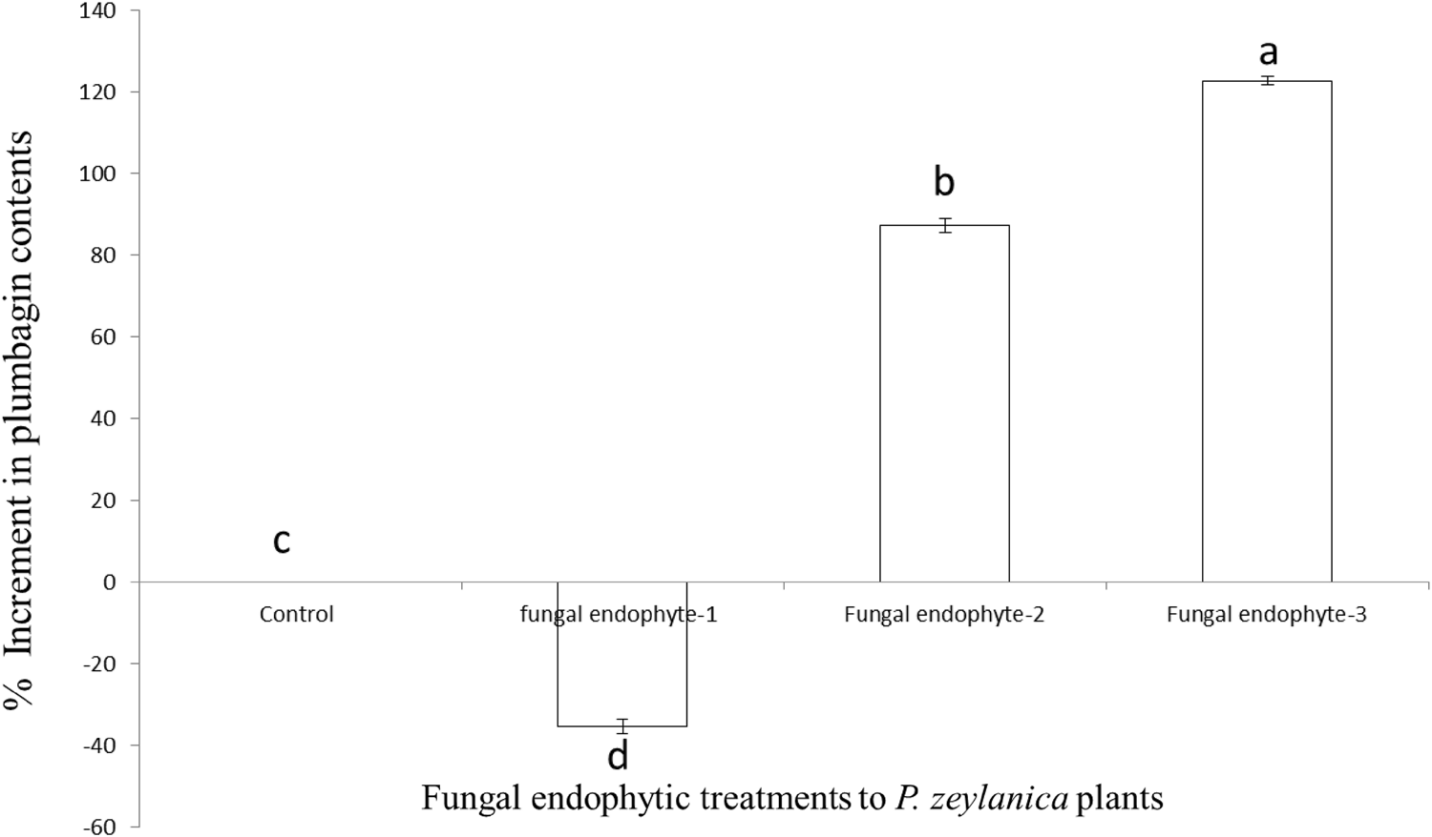
Percent enhancement of plumbagin contents in *Plumbago zeylanica* plants with the treatment of fungal endophytes. ^#^Different letters over the error bars indicate the significant level of difference at 0.05 level of significance

Fungal endophytes reported to influence the synthesis of plumbagin in *P. zeylanica* L. plants after 1 year of the treatment at in vivo level. Among the three fungal endophytes, *Alternaria* sp. (fungal endophytes-3) reported highest increment in the plumbagin contents by 122.68 ± 0.97%. Whereas reduction in the contents of plumbagin contents of the *P. zeylanica* plant treated with *Rhizopus* sp. was also recorded with 35.3618 ± 1.78% (Fig. 1). The concentration of the plumbagin was determined by comparing the LCMS-MS profile of the treated samples with the standard plumbagin profile. The calibration curve obtained from standard Plumbagin with known concentrations is shown in Additional file 2: Figure S1. The chromatogram of standard Plumbagin, the representative chromatograms of the control plant and the treated plants with the respective fungal endophytes are shown in Additional file 2: Figure S1F, respectively. The fungal endophyte, *Aspergillus* sp. (fungal endophytes 2) also reported to enhance the contents of plumbagin (87.17136 ± 1.77% increment) significantly (Fig. 1).

### Characterization of the fungal endophytes

#### Morphological characterization of the fungal endophytes

At preliminary level all the three fungal endophytes were characterised at morphological level (Table 1) at National Fungal Culture Collection of India (NFCCI), Agharkar Research Institute (ARI), Pune (Ref. No. 3/426/2015/Myc, dated 28/04/2015). Based on morphological keys, fungal endophyte 1, fungal endophyte 2 and fungal endophyte 3 were identified as *Rhizopus* sp., *Aspergillus phoenicis* and *Alternaria* sp. respectively.

**Table 1:** Identification of the endophytic fungal isolates of *P. zeylanica*

Sr. no.	Isolate code	Identification at morphological level
1	Fungal endophyte 1	*Rhizopus* sp. Aff. *R. americanus* (Hesselt. & J.J. Ellis) R.Y. Zheng, G.Q. Chen & X.Y. Liu
2	Fungal endophyte 2	*Aspergillus phoenicis* (Corda) Thom & Currie
3	Fungal endophyte 3	*Alternaria* sp.

#### Molecular characterization of the elite fungal endophytes

**Fig. 2:**
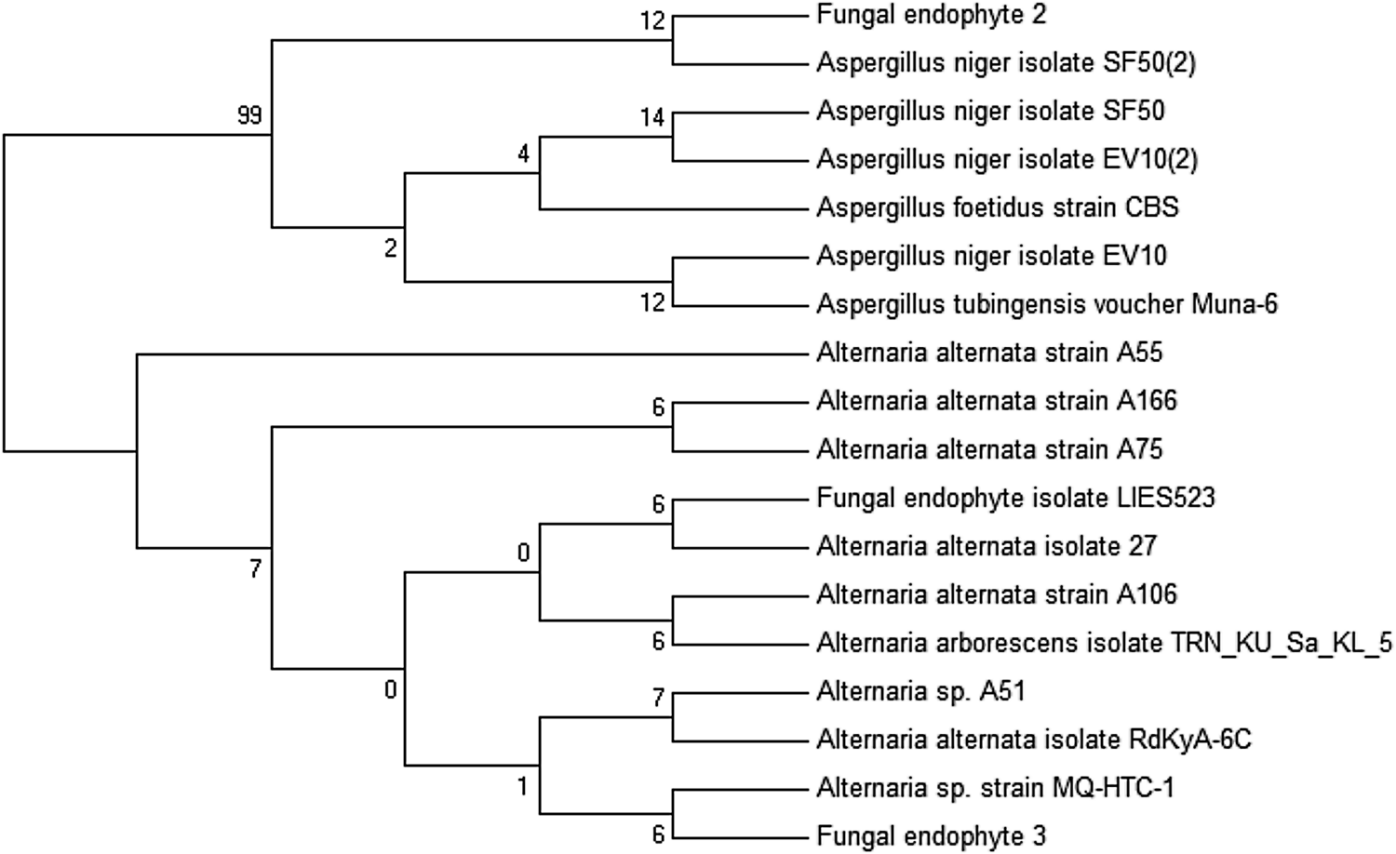
Molecular phylogenetic analysis of the endophytic fungi of *Plumbago zeylanica* by maximum likelihood method along with the homologous ITS sequences retrieved from the gene bank (NCBI)

After morphological characterization, only the elite fungal endophyte (fungal endophyte 2 and fungal endophyte 3) were characterized based ITS gene sequencing technology. The two elite fungal endophytes were identified as *Aspergillus* sp. (fungal endophyte 2) and *Alternaria* sp. (fungal endophyte 3) (Fig. 2).

#### Deposition of the elite fungal endophytes to the microbial repository

The elite fungal endophytes were submitted at NFCCI, ARI, Pune for general deposition and were accessioned as NFCCI-4581 and NFCCI-4582.

## Discussion

Plumbagin is one of the potential anticancer molecules with potential to treat human breast cancer cells (De et al. 2019) and esophageal cancer cells (Cao et al. 2018) etc. and is emerging as a lead pharmaceutical for cancer therapy (Tripathi et al. 2019). As plumbagin is synthesized by the plants of various botanical families in minute quantity, huge quantity of plant material is required to meet the emerging demands of the pharmaceuticals companies, which results in loss of the germplasm if the practice continued with the same pace. So it is needed to enhance the contents of plumbagin using biotechnological approaches (Roy and Bharadvaja 2018). As per literature, the interaction between the fungi and plants leads to enhance the capabilities to synthesise the secondary metabolites efficiently and also enable the plants to withstand in the harsh environmental conditions (Pusztahelyi et al. 2015). As per reports, there are various methods available used to enhance the secondary metabolite in plants; however, technique employing microbes seems most cost-effective. Among the microbes, endophytes have reported to enhance the contents of secondary metabolites efficiently (Maggini et al. 2017).

Two fungal endophytes viz. *Curvularia* sp. CATDLF5 and *Choanephora infundibulifera* CATDLF6 were reported to enhance the contents of vindoline in *Catharanthus roseus* by 229–403% (Pandey et al. 2016). The alteration of secondary metabolites was also reported in *Coleus forskohlii* due to the application of endophytic fungus, *Piriformospora indica* (Das et al. 2012). Almost all plants are considered as the desired host of various endophytes (Kusari et al. 2012). The endophyte enjoys their stay intracellularly or intracellularly in the host plant (Bernardi-Wenzel et al. 2010) without toxicating (Ryan et al. 2008; Lodewyckx et al. 2002), reported to colonise different plant parts (Bernardi-Wenzel et al. 2010), enable the plants to improvise its nutrient uptake, stress tolerance and enhance defence system against the pathogens (Knoth et al. 2014; Rodriguez and Redman 2008; Schulz and Boyle 2005). As per reports various endophytes are reported to synthesise the secondary metabolites similar to the one synthesised by the plants (Kusari et al. 2008; Kusari et al. 2009; Soliman et al. 2011; Vasundhara et al. 2016). Recently an endophytic fungus *Cladosporium delicatulum*, was reported from the leaves and stems of some endemic medicinal plants (*Terminalia pallida*, *Rhynchosia beddomei*, *Pterocarpus santalinus*) of Eastern Ghats region of Andhra Pradesh with potential to produce plumbagin (Venkateswarulu et al. 2018). They further predicted that the interaction of this endophytic fungus and the plant may leads to enhance the contents of plumbagin in plants.

In the present study, we have isolated three endophytic fungi [*Rhizopus* sp. (fungal endophyte 1), *Aspergillus* sp. fungal endophyte 2, and *Alternaria* sp. (fungal endophyte 3] from the roots of the *P. zeylanica.* The plants of the *P. zeylanica* were treated with different fungal endophytes (once a month) and to the control plants only distilled water was added. After completion of 1 year, the plants were harvested and the methanolic root extracts were subjected to LCMS-MS analysis and we have reported highest enhancement of plumbagin contents (122.68 ± 0.97%) in the plants treated with *Alternaria* sp. (fungal endophyte 3). The fungal endophyte *Alternaria alternata* enjoys a vast variety of host plants which belongs to different families viz. *Pinus tabulaeformis* (Guo et al. 2004), *Terminalia pallida* (stem and leaves) (Venkateswarulu et al. 2018), yew tree (inner bark) (Chen et al. 2009), *Taxus cuspidate* (Vasundhara et al. 2016) etc. Depending upon the host of endophytic fungus (*Alternaria* sp.) various types of active biomolecules are reported to be synthesised by this endophytes with important function viz. anticancer, antimicrobial and anti HIV, antidiabetic and anti-oxidant (Eram et al. 2018). We have also reported the enhancement of anticancer molecule (plumbagin) with this *Alternaria* sp. Although we have not checked the production of plumbagin by the said endophytes. This may be due to plant growth promoting nature of the fungal endophyte. Further, the exact mechanism through which these endophytes lead to enhancement of secondary metabolite is still a mystery. Recently, *Cladosporium delicatulum,* an endophytic fungus reported to synthesise plumbagin naturally (Venkateswarulu et al. 2018).

In our study, with another fungal endophyte, *Rhizopus* sp. we recorded reduction in plumbagin content in the *P. zeylanica*. In literature, *Rhizopus stolinifer* is reported as an endophytic fungus with potential to synthesise various secondary metabolites with medicinal importance (Palombo 2011; Faisal et al. 2016), we were expecting enhancement of plumbagin in *P. zeylanica* using *Rhizopus* sp. but we got negative results. Majority of the *Rhizopus* sp. are reported to have pathogenic nature (Ibrahim et al. 2012; Ibrahim and Kontoyiannis 2013) and might be the possible reason for reduction in plumbagin content. Previously, researcher quantified the contents of the plumbagin using TLC, HPTLC (Andhale and Ade 2013) and HPLC (Panichayupakaranant and Tewtrakul 2002; Dorni et al. 2007; Unnikrishnan et al. 2008; Pandey et al. 2013). In the present study, we had employed LCMS-MS tool for the estimation of plumbagin contents in the methanolic root extracts of both control and treated plant. LCMS is the most sophisticated method to detect the contents of the secondary metabolites even at low concentration than other methods of quantification and detection.

The endophytic fungi were characterised at both morphological and molecular level and we have reported change in the identification of the fungal endophytes. During the morphological characterization, fungal endophyte 1, 2, and 3 were identified as *Rhizopus* sp., *Aspergillus phoenicis* and *Alternaria* sp. However based on ITS gene sequencing tool, the elite fungal endophytes were identified as *Aspergillus* sp. (fungal endophyte 2) and *Alternaria* sp. (fungal endophyte 3).

To the best of our knowledge this is the first report on plumbagin enhancement due to endophytic fungi and *P. zeylanica* interaction. Further study is needed to unravel the mechanism associated with the enhancement of plumbagin contents due to interaction of these fungal endophytes.

## Conclusions

The present study reported three fungal endophytes from the roots of *P. zeylanica.* After 1 year the pot-grown plants of *P. zeylanica* reported enhanced production of Plumbagin contents under the influence of the fungal endophytes. Among the three fungal endophytes, maximum enhancement of plumbagin contents (122.68 ± 0.97%) in *P. zeylanica* was recorded with the endophytic fungus (*Alternaria* sp. fungal endophyte 3) at in vivo level.

## Supplementary information

**Additional file 1: Table S1.** Multiple Reaction Monitoring (MRM) Transition of plumbagin.

**Additional file 2: Figure S1**. Estimation of the Plumbagin using LCMS-MS analysis, A: Calibration curve of Plumbagin, B: Chromatogram representing the standard Plumbagin, C: Chromatogram representing the mass and peak of the plumbagin in the methanolic root extract of control plants, D: Chromatogram of representing the mass and peak of the plumbagin in the methanolic root extract of the plants treated with Isolate-1, E: Chromatogram representing the mass and peak of the plumbagin in the methanolic root extract of the plants treated with Isolate-2, F: Chromatogram representing the mass and peak of the plumbagin in the methanolic root extract of the plants treated with Isolate-3.

## Data Availability

Data would be available on request to corresponding author.
