# Supplementary material for: Fungal endophytes of Plumbago zeylanica L. enhances plumbagin content
Source: Bot Stud. 2019 Sep 7;60:21. doi: 10.1186/s40529-019-0270-1 (PMC6732136; doi:10.1186/s40529-019-0270-1)
Supplement: Supplementary file 1 — Additional file 1: Table S1. Multiple Reaction Monitoring (MRM) Transition of plumbagin. [file 40529_2019_270_MOESM1_ESM.docx]

Supplementary information for

**Fungal endophytes of *Plumbago zeylanica* L. enhances plumbagin content**

Namdeo B. Andhale^1,2^ , Mohd. Shahnawaz*^1,3^ and Avinash B. Ade*^1^

^1^Department of Botany, Savitribai Phule Pune University, Ganeshkhind, Pune Maharashtra-411007, India

^2^Department of Botany, Fergusson College, FC Road, Shivajinagar, Pune-411004, MS, India

^3^Present address, Plant Biotechnology Division, CSIR-Indian Institute of Integrative

Medicine, Canal Road Jammu, Jammu-180001, J&K, India

*Corresponding author: Phone: +91-020-25601439. Fax: +91-020-25690498.

Email: mskhakii@unipune.ac.in

avinashade@unipune.ac.in

| Supplementary Table S1. Multiple Reaction Monitoring (MRM) Transition of plumbagin | | | |
| --- | --- | --- | --- |
| Q1 (Precursor) | Q3 (Daughter) | Cone Voltage | Collision Energy |
| 188.95 | 114.81 | 33 | 23 |
| 188.95 | 120.85 | 33 | 16 |
