# Supplementary material for: Fungal endophytes of Plumbago zeylanica L. enhances plumbagin content
Source: Bot Stud. 2019 Sep 7;60:21. doi: 10.1186/s40529-019-0270-1 (PMC6732136; doi:10.1186/s40529-019-0270-1)
Supplement: Supplementary file 2 — Additional file 2: Figure S1. Estimation of the Plumbagin using LCMS-MS analysis, A: Calibration curve of Plumbagin, B: Chromatogram representing the standard Plumbagin, C: Chromatogram representing the mass and peak of the plumbagin in the methanolic root extract of control plants, D: Chromatogram of representing the mass and peak of the plumbagin in the methanolic root extract of the plants treated with Isolate-1, E: Chromatogram representing the mass and peak of the plumbagin in the methanolic root extract of the plants treated with Isolate-2, F: Chromatogram representing the mass and peak of the plumbagin in the methanolic root extract of the plants treated with Isolate-3. [file 40529_2019_270_MOESM2_ESM.docx]

Supplementary information for

**Fungal endophytes of *Plumbago zeylanica* L. enhances plumbagin content**

Namdeo B. Andhale^1,2^ , Mohd. Shahnawaz*^1,3^ and Avinash B. Ade*^1^

^1^Department of Botany, Savitribai Phule Pune University, Ganeshkhind, Pune Maharashtra-411007, India

^2^Department of Botany, Fergusson College, FC Road, Shivajinagar, Pune-411004, MS, India

^3^Present address, Plant Biotechnology Division, CSIR-Indian Institute of Integrative

Medicine, Canal Road Jammu, Jammu-180001, J&K, India

*Corresponding author: Phone: +91-020-25601439. Fax: +91-020-25690498.

Email: mskhakii@unipune.ac.in

avinashade@unipune.ac.in

**
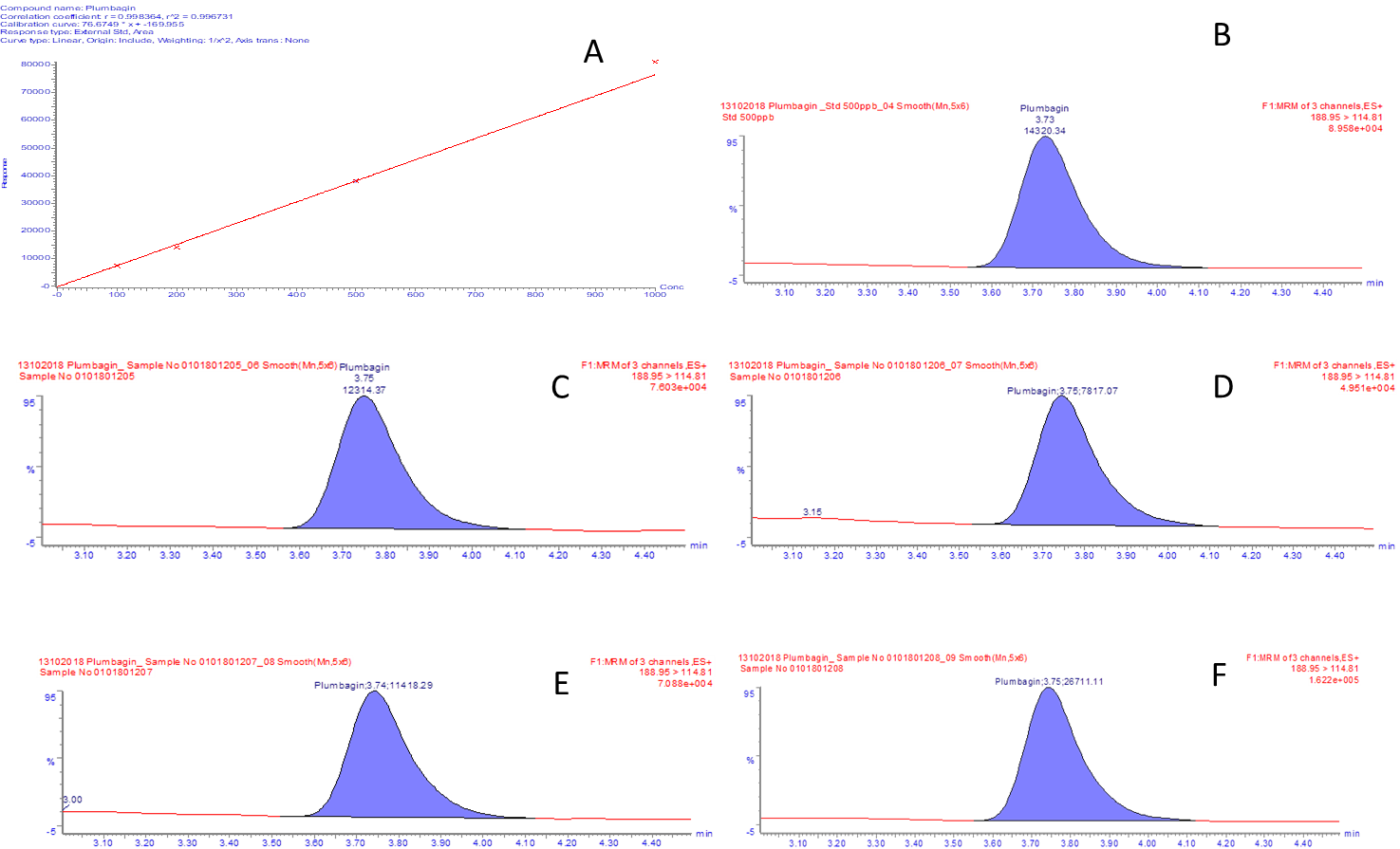
**

Fig. S1.

Fig. S1. Estimation of the Plumbagin using LCMS-MS analysis, A: Calibration curve of Plumbagin, B: Chromatogram representing the standard Plumbagin, C: Chromatogram representing the mass and peak of the plumbagin in the methanolic root extract of control plants, D: Chromatogram of representing the mass and peak of the plumbagin in the methanolic root extract of the plants treated with Isolate-1, E: Chromatogram representing the mass and peak of the plumbagin in the methanolic root extract of the plants treated with Isolate-2, F: Chromatogram representing the mass and peak of the plumbagin in the methanolic root extract of the plants treated with Isolate-3.
